# Propionic Acid Promotes the Virulent Phenotype of Crohn’s Disease-Associated Adherent-Invasive *Escherichia coli*

**DOI:** 10.1016/j.celrep.2020.01.078

**Published:** 2020-02-18

**Authors:** Michael J. Ormsby, Síle A. Johnson, Nuria Carpena, Lynsey M. Meikle, Robert J. Goldstone, Anne McIntosh, Hannah M. Wessel, Heather E. Hulme, Ceilidh C. McConnachie, James P.R. Connolly, Andrew J. Roe, Conor Hasson, Joseph Boyd, Eamonn Fitzgerald, Konstantinos Gerasimidis, Douglas Morrison, Georgina L. Hold, Richard Hansen, Daniel Walker, David G.E. Smith, Daniel M. Wall

**Affiliations:** 1 Institute of Infection, Immunity and Inflammation, College of Medical, Veterinary and Life Sciences, Sir Graeme Davies Building, University of Glasgow, Glasgow G12 8TA, UK; 2 Institute of Biological Chemistry, Biophysics and Bioengineering, Heriot-Watt University, Edinburgh EH14 4AS, UK; 3 Institute for Cell and Molecular Biosciences, Newcastle University, Newcastle-upon-Tyne NE2 4HH, UK; 4 Human Nutrition, School of Medicine, College of Medical Veterinary and Life Sciences, University of Glasgow, Glasgow Royal Infirmary, Glasgow G31 2ER, UK; 5 Scottish Universities Environmental Research Centre, University of Glasgow, Glasgow G75 0QF, UK; 6 Microbiome Research Centre, St George and Sutherland Clinical School, University of New South Wales, Sydney, NSW, Australia; 7 Department of Paediatric Gastroenterology, Hepatology and Nutrition, Royal Hospital for Children, 1345 Govan Road, Glasgow G51 4TF, UK

**Keywords:** adherent-invasive *E. coli*, Crohn's disease, short chain fatty acid, propionic acid

## Abstract

Propionic acid (PA) is a bacterium-derived intestinal antimicrobial and immune modulator used widely in food production and agriculture. Passage of Crohn’s disease-associated adherent-invasive *Escherichia coli* (AIEC) through a murine model, in which intestinal PA levels are increased to mimic the human intestine, leads to the recovery of AIEC with significantly increased virulence. Similar phenotypic changes are observed outside the murine model when AIEC is grown in culture with PA as the sole carbon source; such PA exposure also results in AIEC that persists at 20-fold higher levels *in vivo*. RNA sequencing identifies an upregulation of genes involved in biofilm formation, stress response, metabolism, membrane integrity, and alternative carbon source utilization. PA exposure also increases virulence in a number of *E. coli* isolates from Crohn’s disease patients. Removal of PA is sufficient to reverse these phenotypic changes. Our data indicate that exposure to PA results in AIEC resistance and increased virulence in its presence.

## Highlights

• PA induces virulence-associated changes in CD-associated AIEC
• PA-induced phenotype is reproducible in recently isolated clinical strains
• Phenotypic changes are transcriptional in nature and reversible
• Strains exposed to PA outcompete wild-type strains in a “humanized” murine model

## Graphical Abstract

**Figure:**
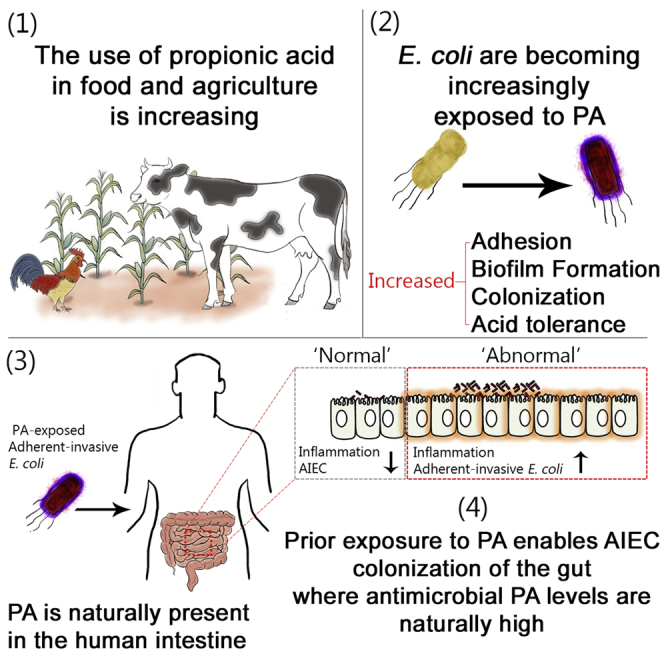

## Teaser

The short chain fatty acid propionic acid is a bacterium-derived human intestinal antimicrobial and immune modulator used widely in Western food production and agriculture. Here, Ormsby et al. demonstrate that exposure to propionic acid induces virulence-associated phenotypic changes in Crohn’s disease-associated adherent-invasive *Escherichia coli* (AIEC).

## Introduction

Short chain fatty acids (SCFAs) are naturally produced by gut bacteria through the breakdown of undigested carbohydrates and starches. This process results in the production of acetic acid (AA), butyric acid (BA), and propionic acid (PA), which together account for approximately 90% of intestinal SCFAs. PA has attracted significant interest due to its potent immunomodulatory effects, with its supplementation shown to reduce the severity of colitis in murine models, suggesting its modulation has potential as a therapeutic intervention in inflammatory bowel disease (Tedelind et al., 2007, Smith et al., 2013). Crohn’s disease (CD) is a debilitating and incurable inflammatory disease of a multi-factorial etiology. The mechanisms underlying the disease are not fully understood; however, it is thought that defects in the immune response to the gut microbiota are a contributing factor (Hansen et al., 2010, Molodecky et al., 2011, Jostins et al., 2012, Mukhopadhya et al., 2012, Imhann et al., 2016, Keestra-Gounder et al., 2016). Sudden changes in diet have been shown to result in rapid changes in the gut microbiota (Turnbaugh et al., 2009, Martinez-Medina et al., 2014, Agus et al., 2016), whereas the inflammation associated with CD results in markedly decreased microbial diversity (Tamboli et al., 2004, Gophna et al., 2006, Frank et al., 2011). Levels of *Enterobacteriaceae* in particular are higher in intestinal samples from CD patients than in healthy controls (Willing et al., 2009, Morgan et al., 2012, Mukhopadhya et al., 2012, Honneffer et al., 2014). One group of *Enterobacteriaceae* that is of particular interest is the *Escherichia coli* pathotype adherent-invasive *E. coli* (AIEC). These bacteria are overrepresented in the ileal microbiota of CD patients, being present in 51.9% of mucosal samples from CD patients compared with 16.7% in healthy controls (Martinez-Medina et al., 2009). Key features of the AIEC pathotype that distinguish them from non-invasive commensal strains include adherence to and invasion of the intestinal epithelium, an increased ability to form biofilms, and the ability to survive and replicate within macrophages without inducing cell death (Martinez-Medina et al., 2009). Although AIEC strains are similar to extra-intestinal pathogenic *E. coli* (ExPEC) in terms of phylogenetic origin and genotype, they have few known virulence factors (Palmela et al., 2018). This apparent lack of virulence factors and the discovery of AIEC strains across all five major diverse phylogroups of *E. coli* mean that an overarching explanation for the origin and virulence of AIEC has remained out of reach.

PA is manufactured on an industrial scale and is now commonly used in agriculture because, in addition to its anti-inflammatory effects, it is a potent antimicrobial. PA has demonstrated success in reducing pathogen numbers in poultry, particularly in reducing *Salmonella* and *Campylobacter* carriage (Hinton and Linton, 1988, Iba and Berchieri, 1995, Hung et al., 2013, González-Fandos et al., 2015). PA is also an effective antimicrobial agent used in Western food production and agriculture, reducing the need for antibiotic use amidst growing antibiotic resistance concerns (Defoirdt et al., 2009, Haque et al., 2012, Khan and Iqbal, 2016). Inclusion in animal feed, grain, and food for human consumption accounted for almost 80% of PA consumption across the world in 2016, with Western Europe (40% of total use), North America (30%), and Asia (23%) as the main consumers (Bizzari and Blagoev, 2013). The success of SCFAs in reducing antibiotic dependence is now seeing their use spread to countries across Africa, the Middle East, and Central and South America (Bizzari and Blagoev, 2013). Although there is increasing evidence for antibiotic-driven enhanced genome-wide mutation rates and horizontal transmission of bacteria from food-producing animals to humans (Levy et al., 1976, Ojeniyi, 1989, Long et al., 2016, Ljubojevic et al., 2017), the role of alternative antimicrobials such as PA in such phenomena has yet to be addressed (Frana et al., 2013, Lazarus et al., 2015, Norizuki et al., 2017). Indeed, using animal models for human disease where SCFAs may play an important role has proven difficult at best. Murine models for human pathogens are limited by distinct differences in basal levels of SCFAs between the murine and human intestines, with murine levels significantly lower in the case of PA (Cummings et al., 1987). However, the significance of such differences in influencing the outcome or course of disease is not known but could be substantial.

In this study, we show that PA exposure promotes increased virulence in AIEC. Using a murine model with increased intestinal PA concentrations, we have generated a more relevant model for human-gut-associated AIEC infection, resulting in increased AIEC virulence and persistence. This increased virulence is PA dependent and can be replicated *in vitro* by exposure of AIEC to PA alone. RNA sequencing (RNA-seq) identified the transcriptional changes driving these changes in virulence, which were determined to be reversible if PA was removed. Our data highlight the potential risks of widespread PA use as an antimicrobial. An understanding of the AIEC phenotype by using genetic markers or phenotypic characteristics has remained elusive; however, our findings indicate that PA metabolism is a crucial driver of the AIEC phenotype.

## Results

### Humanizing the Murine Intestinal PA Concentration Exacerbates the AIEC Phenotype

**Figure 1:**
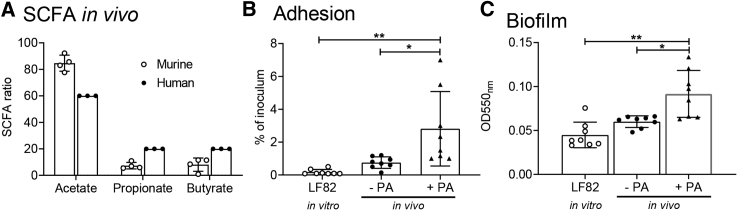
Supplementation of the Murine Intestine with PA Raises the PA Concentration, and Infection of This Model with AIEC Increases the Virulent Phenotype of the Bacterium (A) The ratio of acetate:propionate:butyrate in murine samples was calculated from the isolated caecal contents of an uninfected control mouse that was mock infected with PBS in place of bacteria, 3 days post-treatment with exogenous PA added to the drinking water. This was compared to published human SCFA ratios (Cummings et al., 1987). (B and C) AIEC type strain LF82 modified to contain the luciferase and erythromycin cassette (LF82*lux*) was recovered from mice that had been given water (−PA) or water supplemented with 20 mM propionic acid (+PA). Subsequently, adhesion to Caco-2 intestinal epithelial cells (B) and ability to form biofilms in RPMI with 3% fetal calf serum (FCS) (C) were assessed. *In vitro*/*vivo* refers to where the strains were generated. Results displayed are the average of at least three independent biological replicates ± SD. Samples were analyzed using an un-paired non-parametric t test (A) or one-way ANOVA with Holm-Sidak’s multiple comparisons post-test (B and C; ^∗^p < 0.05; ^∗∗^p < 0.01).

Although the concentration of PA in the human intestine ranges from 1.5 mM in the ileum to 27 mM in the colon, it is considerably lower in the murine intestine (Cummings et al., 1987, Hung et al., 2013). Additionally, the ratios of SCFAs differ greatly (Figure 1A; human AA:PA:BA ratios, 60:20:20; murine ratios, −85:7:8). To address this in our *in vivo* infection model, we supplemented the drinking water of male C57BL6 mice with 20 mM PA. Caecal SCFA levels post-PA supplementation indicated that the relative amount of PA had significantly increased, whereas no significant changes were seen in the relative abundances of either AA or BA (Figure S1; AA:PA:BA ratios without PA, 85:7:8; with PA, 79:12:9). We examined the effect that increased murine intestinal PA levels had on the virulence of the AIEC strain LF82. Mice fed PA-supplemented drinking water for 3 days prior to infection and for the duration of the infection were infected with LF82, alongside control mice that were given only sterile drinking water. Twenty-one days post-infection, LF82 was recovered from the ileum and colon of infected mice. Key phenotypic features that distinguish the AIEC pathotype, adherence to and invasion of the intestinal epithelium, and the ability to form biofilms were then examined (Martinez-Medina et al., 2009). Adhesion to the Caco-2 human intestinal epithelial cell line by LF82 recovered from PA-fed mice was 16-fold higher than wild-type (WT) LF82, whereas no significant change in adhesion was seen in LF82 recovered from mice not fed PA (Figure 1B). There was no significant difference in invasion of LF82 from PA-fed mice (Figure S2; fold change, >5.25). Examination of biofilm formation by LF82 post-*in vivo* infection revealed that anaerobic biofilm formation was dramatically increased in LF82 recovered from mice fed PA relative to the WT strain, whereas there was no significant difference between LF82 recovered from control non-PA-fed mice and the WT strain (Figure 1C).

### The Enhanced AIEC Phenotype Was Driven by PA

**Figure 2:**
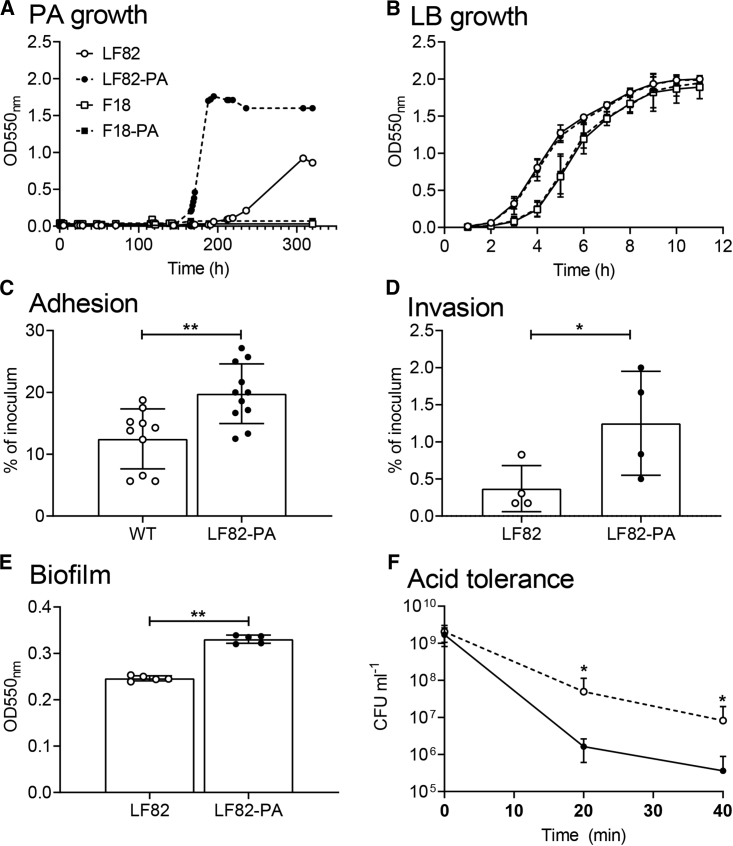
LF82 Utilizes PA as a Sole Carbon Source for Growth, and Exposure to PA Increases Virulence (A) LF82 and commensal *E. coli* strain F-18 were grown in minimal media supplemented with 20 mM PA over a series of five successive re-cultures. Growth rate of the PA-exposed strains, termed LF82-PA and F-18-PA, were subsequently compared to WT controls. (B) The growth rates of LF82, F-18, LF82-PA, and F-18-PA were unchanged in rich LB broth. (C and D) The ability of LF82 and LF82-PA strains to adhere to (C) and invade (D) Caco-2 intestinal epithelial cells was determined. Biofilm formation over 7 days of anaerobic growth was assessed in the presence of 20 mM PA (D). (E) The ability of LF82 and LF82-PA to tolerate acidic pH (pH 3) over time was determined by colony counts (E). Results displayed are the average of at least three independent biological replicates ± SD. Samples were analyzed using an unpaired t test where ^∗^p < 0.05 and ^∗∗^p < 0.01 (C–F).

We hypothesized that the enhanced virulence of LF82 was driven by the increased murine intestinal PA concentration and was independent of other factors during *in vivo* infection. To examine this, LF82 was grown in minimal media with PA as the sole carbon source (20 mM). LF82 was able to grow in PA, whereas a human commensal *E. coli* strain included as a control, *E. coli* F-18 (Ormsby et al., 2016), replicated extremely poorly (Figure 2A). Sub-culturing the bacteria over five growth cycles in PA-supplemented minimal media generated a “PA-exposed” strain of LF82, termed LF82-PA. LF82-PA had a significantly increased growth rate with a doubling time of 3.98 h in PA compared to WT LF82 at 25.59 h (Figure 2A). This increased growth rate was specific to PA and was not observed in nutrient-rich broth (lysogeny broth [LB]; Figure 2B). These results are surprising given the well-documented antimicrobial properties of PA (Hinton and Linton, 1988, Iba and Berchieri, 1995, Hung et al., 2013, González-Fandos et al., 2015).

The effect on virulence of this *in vitro* exposure to PA was examined. LF82-PA showed a significant 1.58-fold increase in adherence to Caco-2 human intestinal epithelial cells compared to LF82 (Figure 2C). Additionally, significantly increased invasion (Figure 2D; 3.38-fold increase) and anaerobic biofilm formation (Figure 2E; 1.34-fold increase) by LF82-PA was observed compared to LF82. PA exposure had no significant effect on the intracellular replication of LF82 (Figure S3).

Direct incorporation of PA into the membrane is a mechanism used by bacteria to minimize the toxic effects of excess PA in the environment (Jain et al., 2007, Lee et al., 2013, Si et al., 2016). Given that the phenotypic changes seen in AIEC, such as increased adhesion, were likely to be mediated by changes in the composition of the bacterial membrane following growth on PA, we investigated this further. Gas chromatography coupled to isotope ratio mass spectrometry (GC-IRMS) using ^13^C-labeled PA (1-^13^C sodium propionate) revealed that PA was not incorporated into odd chain long chain fatty acids (LCFAs). However, there was significant ^13^C-enrichment in 12 fatty acid methyl esters (FAMEs) that did not correspond to any of the 37 FAMEs in our reference standard. The proximity of these labeled peaks to known LCFAs is likely indicative of incorporation of PA into methylated or branched chain fatty acids (BCFAs), as described previously during *Mycobacterium tuberculosis* growth on, and detoxification of, PA (Lee et al., 2013). Therefore, this indicated that LF82 could both metabolize and detoxify an antimicrobial that exerts potent toxic effects on a number of other intestinal pathogens (Hinton and Linton, 1988, Iba and Berchieri, 1995, Hung et al., 2013, González-Fandos et al., 2015).

The observed changes in the bacterial membrane also rendered LF82-PA increasingly acid tolerant despite exposure in PA-supplemented minimal media being carried out at pH 7.4 (Figure 2F). A reduction in cell number was seen for both LF82 and LF82-PA at a pH of 3, but the LF82-PA strain survived in greater numbers for longer periods (at 20 min, LF82-PA was recovered in numbers >30.4-fold higher than LF82; at 40 min, LF82-PA was >22.7-fold higher). Taken collectively, these results indicate that the increased virulence observed after passage of LF82 through a PA-supplemented murine model can be replicated by exposure to PA *in vitro*.

### The Enhanced Virulence Phenotype of LF82-PA Is Not Genome Encoded and Is Reversible

**Figure 3:**
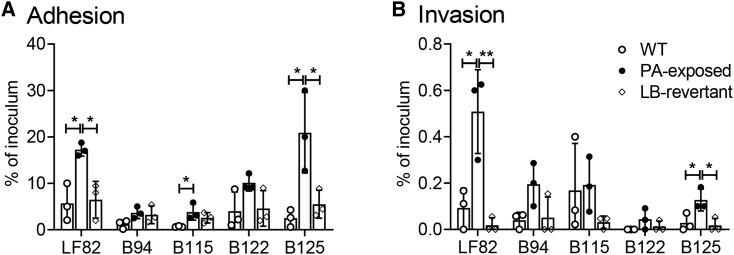
The Enhanced Fitness and Virulence of the AIEC-PA Phenotype Can Be Reversed AIEC type strain, LF82, and clinical isolates B94, B115, B122, and B125 were repeatedly cultured in minimal media with 20 mM PA as the sole carbon source. These isolates were then continuously re-cultured (5 passages) in LB, generating a reverted strain (termed Isolate-PA-LB). Adhesion (A) and invasion (B) to Caco-2 intestinal epithelial cells were examined. Data displayed are of two independent experimental replicates, with each experiment including three independent biological replicates. Adherence and invasion data are expressed as mean ± SD; data were analyzed using a one-way ANOVA with Tukey post-test correction (^∗^p < 0.05; ^∗∗^p < 0.01).

Genome sequencing of three biological replicates of LF82-PA, exposed independently *in vitro* to PA, revealed a number of single nucleotide polymorphisms (SNPs; Data S1, nucleotide analysis of PA-exposed LF82-PA, related to Figure 2). However, no SNPs were conserved across all isolates. Detailed analysis of the genes and pathways in which the SNPs were identified did not lead to the identification of any candidate pathways that may explain the changes in virulence observed. However, we cannot exclude the possibility that different combinations of small genomic changes may result in the same outcome at the transcriptional level. As the virulent phenotype persists over a number of generations and was not explained by genetic analysis, we hypothesized that the phenotype we see may be as a result of an epigenetic switch in LF82-PA. A long-term epigenetic memory switch with a role in controlling bacterial virulence bimodality was recently identified in enteropathogenic *E. coli* (EPEC) (Ronin et al., 2017). This “resettable phenotypic switch” results in populations of virulent and hypervirulent genetically identical subpopulations that are retained through generations. To further examine this possibility, LF82-PA was passaged through rich (LB) media with no PA-selective pressure. After five successive subcultures, this strain (LF82-PA-LB) had lost its increased growth rate in PA, and its virulence phenotype reverted to be more similar to that of WT LF82 (Figure 3). The epigenetic nature of this change was further confirmed through sequencing of the reverted LF82-PA-LB strains, which indicated that the SNPs present in the original LF82-PA strains were conserved and that the changes induced by PA were not due to SNPs or mutations (Data S1).

### The PA-Driven Enhanced AIEC Phenotype Is Seen in Other Clinical AIEC Isolates

*E. coli* isolated from intestinal samples of pediatric patients with active CD was compared to LF82 for its ability to adhere to and invade an intestinal epithelial cell line before and after exposure to PA (Figure 3). All isolates exhibited an AIEC phenotype with an ability to adhere to and invade intestinal epithelial cells (Ormsby et al., 2019). Although there was an increase in the ability of all isolates to adhere to intestinal epithelial cells after PA exposure, this was only significant for clinical isolates B115 and B125 (Figure 3A). A similar increase was observed for invasion (Figure 3B). However, the phenotype was reversible in the same manner as previously for LF82 through removal of the PA pressure. Isolates were grown in rich media containing no PA for five successive growth cycles before re-examination of their ability to adhere to and invade intestinal epithelial cells, with all strains examined returning to WT levels of adhesion and invasion (Figure 3). These data indicated that PA-induced exacerbation of the AIEC phenotype occurred more widely in AIEC isolated from CD patients.

### Enhanced LF82-PA Virulence Is Driven by Transcriptional Changes

**Figure 4:**
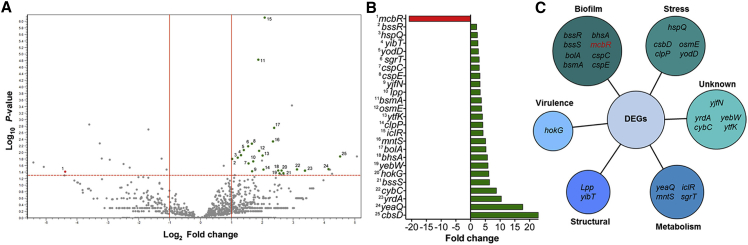
Global Transcriptional Shifts of LF82-PA (A) Volcano plot illustration of gene expression between LF82 and LF82-PA, as determined by RNA-seq. Significance (Log_10_ p value) and fold change cutoffs (log_2_) are indicated by the dashed and solid lines, respectively. (B) Significantly differentially expressed genes (DEGs) are numbered and highlighted in green (upregulated in LF82-PA) and red (downregulated). (C) Genes corresponding to ribosomal RNA coding regions were not labeled for clarity. Bar chart highlighting the fold changes in expression of the identified DEGs. Each gene is numbered and corresponds to the volcano plot. DEGs are grouped into functional categories.

Given that no definitive mutational basis for the observed increase in virulence was detected through genome analysis, we used a comparative RNA-seq approach to probe the global transcriptional profiles of LF82 and LF82-PA grown on PA. RNA-seq revealed 25 differentially expressed genes (DEGs; p ≤ 0.05) between LF82 and LF82-PA (Figure 4; Table S2); 24 were upregulated in the LF82-PA strain and 1 (*mcbR*; −20.85-fold) was downregulated. Of the 25 DEGs identified by RNA-seq, 21 including *mcbR*, were validated as significantly altered by PA using qRT-PCR (Figure S4). Functional grouping of these 21 DEGs revealed their roles in diverse processes, including biofilm formation, stress responses, metabolism, membrane integrity, and transport of alternative carbon sources (Figure 4; Table S1). Eight DEGs have well-described roles in biofilm formation, further adding to our *in vitro* findings indicating that PA was a driver of adhesion and biofilm formation (Figures 2C and 2E). Upregulation of another DEG, a regulator of membrane fatty acid composition *yibT*, adds further evidence for the potential detoxification of PA through membrane incorporation, as previously described (Lee et al., 2013, Si et al., 2016). Therefore RNA-seq analysis indicates that PA drives changes in virulence that are fundamental to the AIEC pathotype.

### Exposure to PA *In Vitro* Significantly Increases Persistence of LF82 *In Vivo*

**Figure 5:**
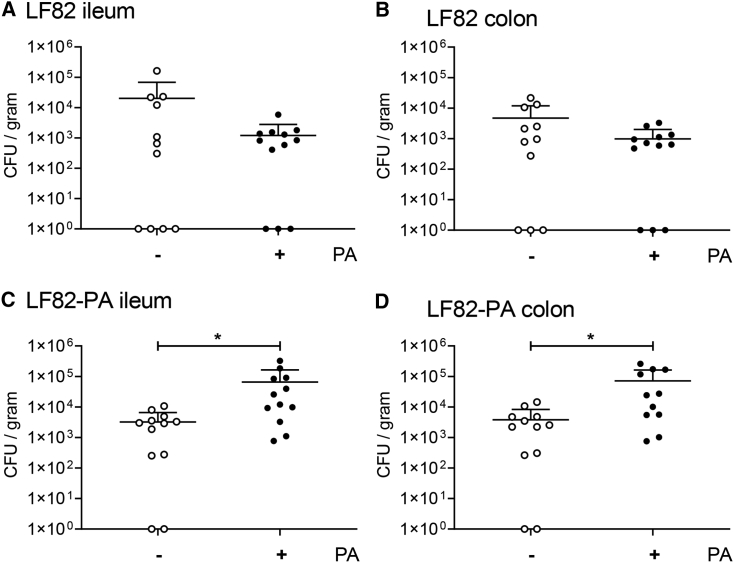
Pre-exposure of AIEC to PA Combined with Exogenous PA Supplementation Promote Colonization and Long-Term Persistence Drinking water was supplemented where indicated with 20mM PA and provided to male C57BL/6 mice for 3 days prior to infection. Persistence of LF82 (A and B) and LF82-PA (C and D) was determined in the ileum (A and C) and colon (B and D) 21 days post-infection by colony counts. Data are expressed as CFU/gram of tissue ± SD and were analyzed using an unpaired t test (^∗^p < 0.05).

Given our findings of PA-driven changes in virulence, we determined the effect of PA exposure on long-term persistence of LF82-PA *in vivo*. Mice were again provided PA-supplemented (20 mM) water for 3 days prior to infection and for the 21-day duration of the infection. Mice given only sterile drinking water and infected with LF82 or LF82-PA or those treated with phosphate-buffered saline (PBS) were included as controls. In PA-fed mice, the colonization of LF82 was not significantly altered in either the ileum or colon compared to control mice (Figures 5A and 5B). However, LF82-PA was found to persist with a greater than 20-fold increase in the ileum and a greater than 18-fold increase in the colon in these mice compared to controls (Figures 5C and 5D). No significant difference in the persistence of either strain was observed in the caecum, irrespective of the presence of PA (Figure S5). These data indicate that PA-exposed strains retain virulence when transferred to a new model host, most likely through having an increased nutritional advantage as well as an increased ability to adhere to and invade intestinal cells and form biofilms.

### Exposure to PA *In Vivo* Gives LF82 a Competitive Advantage *In Vivo*

**Figure 6:**
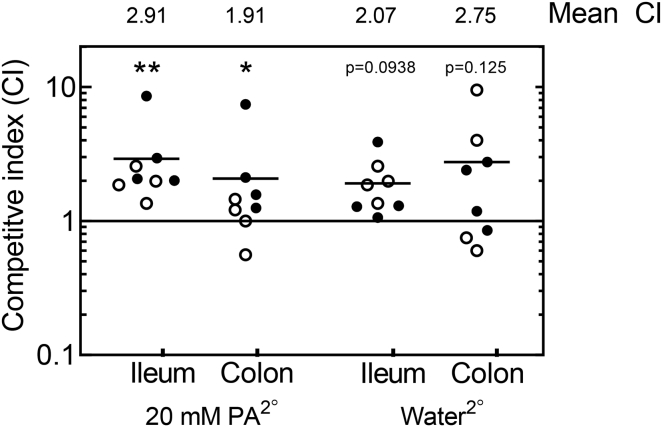
Competitive Index of LF82 from PA-Fed Mice versus LF82 from Water-Fed Mice For the primary infection (1°), one group of mice (n = 4) were fed 20 mM PA for 3 days prior to challenge. A second group of mice were given only sterile water. A subset of each of these mice were then infected with either LF82 or LF82 carrying a luciferase and erythromycin cassette (LF82*lux*), and the infection was allowed to proceed for 7 days. This resulted in the recovery of strains labeled LF82-PA^1°^, LF82-W^1°^, LF82*lux*-PA^1°^, and LF82*lux*-W^1°^ from ileal and colonic homogenates. Bacteria were isolated using LB supplemented with either ampicillin or erythromycin. Subsequently, equal quantities of LF82-PA^1°^ and LF82*lux*-W^1°^ were mixed, as were LF82*lux*-PA^1°^ and LF82-W^1°^, giving final concentrations of 1 × 10^9^ CFU mL^−1^ (0.5 × 10^9^ CFU mL^−1^ of each strain). The ratios of LF82-PA^1°^:LF82*lux*-W^1°^ and LF82*lux*-PA^1°^:LF82-W^1°^ were determined by plating on LB plates supplemented with ampicillin or erythromycin. Mice were again given either 20 mM PA or sterile drinking water for 3 days prior to challenge, before infection with either LF82-PA^1°^:LF82*lux*-W^1°^ or LF82*lux*-PA^1°^:LF82-W^1°^. Seven days post-infection, bacteria were recovered by plating ileal and colonic homogenates on either ampicillin or erythromycin. Competitive indices (CI) were determined by normalization to the initial inoculum ratios. Black circles represent LF82-PA^1°^, whereas white circles represent LF82*lux*-PA^1°^. The solid black line at CI = 1 represents LF82-W^1°^ and LF82*lux*-W^1°^. Statistical analyses for each dataset were conducted using the individual colony counts. A two-tailed Wilcoxon rank-sum test was conducted (^∗^p < 0.05 was deemed significant).

As exposing LF82 to PA *in vivo* resulted in strains with an increased ability to adhere to and invade intestinal epithelial cells and form biofilms *in vitro* (Figures 1B and 1C), we examined the capacity of these strains to outcompete LF82 that had not been exposed to increased PA. LF82 and LF82*lux* were used to infect mice supplemented with either PA or sterile drinking water, before being recovered. A competiton assay using equal mixtures of these re-isolated strains was then conducted in two subgroups of mice: one whose diet was supplemented with PA and a second whose diet was not. Our data indicated that LF82 recovered from a primary PA (PA^1°^)-fed mouse outcompeted bacteria from a primary water (W^1°^)-fed mouse in subsequent infections of both PA and water-fed secondary mice (PA^2°^ and W^2°^, respectively) (Figure 6). PA^1°^-recovered bacteria outcompeted W^1°^-recovered bacteria in the ileum (competitive index [CI] = 2.91; p = 0.0078) and colon (CI = 1.91; p = 0.0156) of a PA^2°^-fed mouse. In a W^2°^ mouse, PA^1°^-recovered bacteria outcompeted W^1°^-recovered bacteria in the ileum (CI = 2.07; p = 0.0938) and colon (CI = 2.75; p = 0.125).

## Discussion

SCFAs have significant effects on their hosts and are seen as key players in how the intestinal microbiome maintains health, modulates the immune system, controls invading pathogens, and even exerts effects on distal sites such as the brain (Smith et al., 2013, Jacobson et al., 2018, van de Wouw et al., 2018). Such positive effects have led to the suggested use of SCFAs as a therapeutic intervention strategy in inflammatory diseases, including inflammatory bowel disease (Tedelind et al., 2007). Here, however, we have shown a role for the SCFA PA in microbial infection, acting as a driver for virulence of the bacterial pathotype AIEC that is commonly isolated from the intestine of CD patients (Martinez-Medina et al., 2009). Exposure of AIEC to PA, in contrast to other pathogens where it is a negative regulator of virulence, stimulated adhesion and biofilm formation and induced an upregulation of an array of genes related to virulence.

Both the antimicrobial properties of PA and its negative regulation of bacterial virulence are the basis for its widespread use in agriculture as PA clears *Salmonella* and *Campylobacter* spp. rapidly post-treatment of poultry (Hinton and Linton, 1988, Iba and Berchieri, 1995, Hung et al., 2013, González-Fandos et al., 2015). As well as its direct toxicity to these pathogens, this inhibitory effect is mediated by negative regulation of genes critical for intestinal colonization and is thought to be a response by these pathogens to the varying PA concentrations in the human intestine. In *Salmonella*, high concentrations of PA undermine the stability of virulence regulators, such as HilD, meaning *Salmonella* is less likely to colonize the lower intestine where PA levels are highest (Hung et al., 2013). In direct contrast, we have shown here that PA positively regulates virulence of the AIEC strain LF82. Increasing exposure to PA, and increasing concentrations in the murine intestine, results in a >20-fold increase in persistence as well as increasing the most notable phenotypic traits of AIEC, such as adhesion and invasion of the intestinal epithelium and biofilm formation. There are limited bacteria to draw comparisons to with regard to PA and virulence, as few respond positively to PA given its antimicrobial properties. Enterohemorrhagic *E. coli* (EHEC) and *Citrobacter* increase virulence by the type three secretion system (T3SS) in response to PA, as previously shown (Nakanishi et al., 2009, Connolly et al., 2018). However, *Mycobacteria* do become increasingly virulent in the presence of PA with overlapping strategies used by mycobacteria and AIEC as both metabolize and directly incorporate PA into their membrane lipids (Jain et al., 2007, Upton and McKinney, 2007, Lee et al., 2013, Si et al., 2016). This strategy of PA incorporation into the outer membrane of AIEC, as well as detoxifying PA, also increases resistance to pH and likely plays a role in the increased adhesion and invasion noted with LF82 after PA exposure (Figure 2).

Although PA has significant immunomodulatory properties in the intestine, disease context is highly important. Although PA supplementation in certain murine models reduces disease through signaling to specific immune cells, here, PA supplementation in the presence of AIEC resulted in significant overgrowth of the bacteria due to PA-driven phenotypic switches that occurred (Smith et al., 2013). The dramatic increase in colonization with increasing PA in the murine intestine also highlights a significant problem with murine models of intestinal disease. PA levels, along with those of other SCFAs, are significantly different in the murine intestine compared to the human intestine, which is likely a contributing factor in the very different outcomes in bacterial infection in these hosts (Cummings et al., 1987). Although differing microbiomes are a factor in susceptibility to infection, it is probable that these protective effects of the microbiome are mediated through, and dependent on, the production of SCFAs and other antimicrobial molecules by the intestinal microbiota (Jacobson et al., 2018). Interestingly, in this regard, the caecum where the majority of SCFAs are produced in the murine intestine was distinct from the ileum and colon during infection, as no significant increase in colonization by AIEC was detected here. It is possible that the levels and types of antimicrobials being produced in the caecum still proved refractory to increased AIEC colonization despite its PA adaptation.

Our ability to recapitulate *in vitro* the effects of PA on AIEC indicates that PA in isolation exerts a significant effect on bacterial virulence. This was further demonstrated using clinical isolates of *E. coli* derived from CD patients that were exposed to PA and tested for their ability to adhere to and invade human colonic epithelial cells. Although the clinical isolates were from the intestine of CD patients, it is unknown if they are true AIECs given the confusion over what constitutes the AIEC pathotype (Elhenawy et al., 2018). However, our phenotypic examination of these isolates suggests that they are likely to be AIEC (Figure 3). Exposure to PA induced significant increases in virulence in all clinical strains. In comparison, the human commensal isolate F-18 was not able to adapt to PA. These data suggest that certain *E. coli* isolates recovered from the human CD intestine are readily adaptable to PA and that contrary to its effects on other pathogens, PA is actually a driver for AIEC virulence and does not exert antimicrobial effects. Further analysis of the PA effect on a large range of *E. coli* pathobionts is necessary to definitively determine if this effect is AIEC specific.

Here, we have shown through our work using *in vivo* models that LF82 isolated post-PA supplementation in a murine model is more virulent than without such treatment. This indicates that rather than being directly inhibited by the antimicrobial effects of PA, these strains instead show potential to be readily adaptable to the naturally higher concentrations of PA in the human intestine. Given the wide use of PA environmentally and agriculturally, it is not inconceivable that bacteria such as AIEC come into contact with such concentrations of PA, as those in animal water, feed, and silage are reported to be 20 mM and higher (Hinton and Linton, 1988, European Food Safety Authority., 2013, Hung et al., 2013, González-Fandos et al., 2015) Such exposure would likely make the PA concentrations in the human intestine, which increases from 1.5 mM in the ileum to 27 mM in the colon, easily tolerable to *E. coli* strains as we have indicated here (Cummings et al., 1987, Hung et al., 2013). Horizontal transmission of strains from poultry to humans as previously seen, driven by antibiotics, would therefore seem highly possible. Additionally, recent evidence has suggested the food additive trehalose is a contributory factor in the emergence and hypervirulence of two epidemic lineages of *Clostridioides difficile* (Collins et al., 2018). Therefore, although the focus rightly remains on antibiotic resistance, more work is needed to determine the long-term effects of alternative antimicrobials in generating more resistance and more virulent bacteria capable of horizontal transmission. Although, in addressing one resistance problem, we must be careful that another is not inadvertently created.

Most encouragingly, we were able to show that the *in vitro* and *ex vivo* phenotypes that we observed were directly relatable to increased *in vivo* virulence (Figure 6). Our competitive assay between isolates passaged through WT mice and mice with a PA-supplemented diet revealed that those isolates that were exposed to PA *in vivo* were able to outcompete those that were not, in a PA-fed secondary mouse. Although in a water-fed secondary mouse this out-competition was not significant, there was a still an observed trend toward those isolates initially recovered from a PA-fed mouse. However, this recapitulates the *in vitro* observations made previously, in that the PA -phenotype is reversible with the removal of PA selective pressure.

Our findings here are not without precedent. Dietary additives and a mock Western diet have been demonstrated to contribute to increased colonization of AIEC in murine models (Martinez-Medina et al., 2014, Agus et al., 2016). This work explores the finding of PA as a paradoxical pro-virulence factor in AIEC, which is at odds with its perceived role as an antimicrobial. The growing use of PA in the Western diet, coupled with the rapid expansion of CD incidence in recent years, highlights the importance of this work in suggesting a potential mechanism for diet as a key driver of selection in the gut, which would favor the carriage and transformation of an emerging pathogenic *E. coli* variant.

## STAR★Methods

### Key Resources Table

**Table:** 

REAGENT or RESOURCE	SOURCE	IDENTIFIER
**Bacterial and Virus Strains**		

LF82: Wild-type	Prof. Daniel Walker, Uni. of Glasgow	LF82
LF82-PA.1 (LF82 exposed to 20 mM PA; rep 1)	*This study*	LF82-PA.1
LF82-PA.2 (LF82 exposed to 20 mM PA; rep 2)	*This study*	LF82-PA.2
LF82-PA.3 (LF82 exposed to 20 mM PA; rep 3)	*This study*	LF82-PA.3
LF82Δ*eutR*: LF82 with *eutR* knocked out	*This study*	LF82Δ*eutR*
LF82-PAΔ*eutR*: LF82-PA.1 with *eutR* knocked out	*This study*	LF82-PAΔ*eutR*
F-18	Ormsby et al., 2016	F-18
B94	UK Clinical Research Network (9633)	B94
B115	UK Clinical Research Network (9633)	B115
B122	UK Clinical Research Network (9633)	B122
B125	UK Clinical Research Network (9633)	B125

**Chemicals, Peptides, and Recombinant Proteins**		

Lysogeny Broth (LB) media	LabM	NCM0088A
Bacto Agar	Formedium	AGA02
Sodium chloride	Merck	N/A
Ammonium chloride	Fisher Scientific	12125-02-9
Potassium hydrogen phosphate	Merck	7758-11-4
Trace metal solution	Cold Spring harbor protocols	N/A
Magnesium sulfate	VWR Chemicals	7487-88-9
Calcium chloride	Fisher Scientific	22189-08-8
Thiamine hydrochloride	Fisher Scientific	67-03-8
Iron chloride	Fisher Scientific	10025-77-1
Ethylenediaminetetraacetic Acid (EDTA)	Fisher Scientific	60-00-4
Taurocholic acid	Fisher Scientific	345909-26-4
D-glucose	Sigma	50-99-7
Sodium propionate	Sigma	137-40-6
1,2-Propanediol	Sigma	57-55-6
Ethanolamine	Sigma	141-43-5
RPMI-1640	Thermofisher	31870025
Foetal calf serum	Fisher Scientific	11573397
L-glutamine	Fisher Scientific	15430614
No-Carbon-E (NCE media)	Cheng et al., 2011	N/A
Cyano-cobalamin	Fisher Scientific	68-19-9
RNAprotect	QIAGEN	76526
RNAlater	Thermofisher	10391085
Penicillin/streptomycin	Sigma	P4333
Triton X-100	Merck	T9284
Acetic Acid	VWR Chemicals	20104.334
Crystal Violet	Merck	C0775
M9 Minimal Salts, 5x	Merck	M6030
Dulbeccos Modified Eagle Medium (DMEM)	Sigma	D5671
Foetal Bovine Serum, Heat Inactivated	Invitrogen	10500064
Phosphate Buffered Saline, PBS	Invitrogen	14190086
Streptomycin Sulfate Salt	Sigma	S9137
Erythromycin	Sigma	E5389
Ampicillin	Sigma	A1593
Gentamcin	Merck	G1264
LPS from Salmonella Typhimurium	Merck	L7770
Hydrochloric Acid	Merck	258148
Turbo DNase	Thermofisher	AM2238

**Critical Commercial Assays**		

Affinity Script cDNA multi-temp Synthesis Kit	Agilent	26000-50
PerfeCTa SYBR Green FastMix	Quanta Biosciences - VWR	200436
MicrobeExpress mRNA Kit	Invitrogen	AM1905
RNEasy Mini Kit	QIAGEN	74104

**Deposited Data**		

RNA-seq and Genomic sequence data	This paper	ENA:PRJEB36206

**Experimental Models: Cell Lines**		

Caco-2 Human Intestinal Epithelial cells	American Type Culture Collection (ATCC)	ATCC HTB-37
RAW264.7 murine macrophage cell line	American Type Culture Collection (ATCC)	ATCC TIB71

**Experimental Models: Organisms/Strains**		

Mouse C57BL/6J (Male; 6-8 Weeks old)	Envigo	N/A

**Oligonucleotides (5′-3′)**		

Oligonucleotides can be found in Table S1	Sigma	N/A

**Software and Algorithms**		

GraphPad Prism v7.0c	https://www.graphpad.com	N/A
Mascot search engine v2.6.2	http://www.matrixscience.com	N/A
MAUVE v2.4.0	http://darlinglab.org/mauve/mauve.html	N/A
CLC Genomics Workbench v7.0.1	https://digitalinsights.qiagen.com/	N/A
ExPASy	https://www.expasy.org	N/A
EMBOSS Needle	https://www.ebi.ac.uk/Tools/psa/emboss_needle	N/A

**Other**		

Breathe-Easy Sealing Membrane	Merck	2380059-1PAK

### Lead Contact and Materials Availability

Further information and requests for resources and reagents should be directed to and will be fulfilled by the Lead Contact, Dr Daniel M. Wall (Donal.Wall@glasgow.ac.uk). All unique reagents generated in this study are available from the Lead Contact with a completed Materials Transfer Agreement.

### Experimental Model and Subject Details

The Caco-2 human intestinal epithelial cell (IEC) line obtained from the American Type Culture Collection (ATCC) was maintained in Dulbecco’s Modified Eagle Medium (DMEM) medium (Sigma) supplemented with 10% Heat-inactivated FBS (Sigma), L-glutamine and penicillin/streptomycin (Sigma).

#### RAW 264.7 murine macrophages

RAW 264.7 macrophages were obtained from the ATCC and maintained in Roswell-Park Memorial Institute (RPMI) media supplemented with 10% Fetal bovine serum (FBS), L-glutamine and penicillin/streptomycin (Sigma). Cells were maintained at 37°C and 5% CO_2_ with regular media changes.

#### Animal experiments

All animal procedures were approved by an internal University of Glasgow ethics committee and were carried out in accordance with the relevant guidelines and regulations as outlined by the UK Home Office (PPL 70/8584). Male C57BL/6 mice aged between eight and ten weeks were obtained from The Jackson Laboratory (Envigo). Twenty millimolar sodium propionate was administered to C57BL/6 mice in drinking water three days prior to infection. Control mice were given only sterile water. Twenty-four hours prior to infection, mice were treated with an oral dose of 20 mg streptomycin before oral infection with 0.1 mL PBS (mock-infected) or with approx. 1 × 10^9^ colony forming units (CFU) of LF82 (*lux*) or LF82-PA (*lux*). Mice were euthanized 3 days after infection for colonization experiments and 21 days after infection for persistence experiments, with caecal contents collected for SCFA analysis. Ileal, caecal and colonic tissue were weighed and homogenized for enumeration of bacterial numbers. Bacterial numbers were determined by plating tenfold serial dilutions onto LB agar containing the appropriate antibiotics. After 24 h of incubation at 37 °C, colonies were counted and expressed as CFU per gram of tissue.

#### *In vivo* competition assay

Male C57BL/6 mice aged between eight and ten weeks were obtained from Envigo. Twenty millimolar sodium propionate was administered to C57BL/6 mice in drinking water three days prior to infection. Control mice were given only sterile water. Twenty-four hours prior to infection, PA-treated and control mice were given an oral dose of 20 mg streptomycin before oral infection with 0.1 mL PBS (mock-infected) or with approx. 1 × 10^9^ colony forming units (CFU) of LF82 or LF82*lux*. After 7 days of infection, mice were culled and bacteria recovered from both the ileum and colon on LB containing ampicillin (LF82) or erythromycin (LF82*lux*). These strains were hence termed LF82-PA^1°^; LF82-W^1°^; LF82*lux*-PA^1°^; or LF82*lux*-W^1°^, respectively. Next, equal quantities of LF82-PA^1°^ *and* LF82*lux*-W^1°^ were mixed, as were LF82*lux*-PA^1°^ and LF82-W^1°^, giving final concentrations of approximately 1 × 10^9^ CFU ml^-1^ (0.5 × 10^9^ CFU ml^-1^ of each strain). The ratios of LF82-PA^1°^:LF82lux-W^1°^ and LF82*lux*-PA^1°^:LF82-W^1°^ were determined by plating on LB plates supplemented with ampicillin or erythromycin. Mice were challenged as previously bacteria recovered through plating of ileum and colon homogenates on LB agar supplemented with ampicillin or erythromycin. Competitive indices were determined by normalization to the initial inoculum ratios.

### Method Details

#### Bacterial strains and growth conditions

Pathogenic AIEC strain LF82 and intestinal commensal *E. coli* strain F-18 were used in this study and were cultivated on Lysogeny broth or agar. M9 minimal medium supplemented with 20 mM PA (M9-PA [20% M9 salts (32 g Na_2_H_2_PO_4_2H_2_O (Merck), 12.5 g NaCl (Merck), 2.5 g NH_4_Cl (Fisher scientific), 7.5g KH_2_PO_4_ (Merck) and 400 mL H_2_O], 0.1% Trace metal solution, 0.2 mM MgSO_4_ [VWR chemicals], 0.02 mM CaCl_2_ [Fisher scientific], 1 mM Thiamine, 0.01% 5 g/L FeCl_3_, 0.01% 6.5 g/L EDTA, 0.1% taurocholic acid, 20 mM Sodium propionate and dH_2_O) was used for growth. Strains were grown in 100 mL of M9-PA at 37°C at 180 rpm, unless stated. Bacterial growth was measured at optical density 600nm (OD_600nm_). To obtain adapted cells, upon reaching stationary phase, cultures were back-diluted into fresh M9-PA. Strains for infection were back-diluted after overnight growth into 10 mL cultures of RPMI-1640 (Sigma) supplemented with 3% fetal calf serum (FCS) and L-glutamine. These were then grown at 37°C in a shaking incubator at 180 rpm to an OD_600nm_ of 0.6 before further dilution to give final multiplicities of infection (MOI) of 10 or 100. Real-time PCR was conducted using bacteria grown in No-Carbon-E (NCE) media (Davis et al., 1980). Twenty millimolar sodium propionate (Sigma), 1,2-propanediol (Fisher Scientific) or D-glucose (Sigma) were added with 200 nM cyano-cobalamin (Sigma) to act as an electron acceptor (Price-Carter et al., 2001). Cultures were grown overnight in LB, washed three times in NCE media with no carbon source added, and inoculated 1:100 into 10 mL NCE media containing each respective carbon source. Cultures were grown until mid-log phase (OD_600_ of 0.6) and used for RNA-extraction.

Clinical isolates (B94, B115, B122 and B125) were from the “Bacteria in Inflammatory bowel disease in Scottish Children Undergoing Investigation before Treatment” (BISCUIT) study (Hansen et al., 2013). Isolates B94, 115, 122 and 125 were recovered from patients with Crohn’s disease. The median (range) age was 13.7 (11.2 to 15.2), height z-score was −0.4 (−2.0 to 0.2), weight z-score was −0.7 (−3.4 to −0.1), and BMI z-score was −1.3 (−4.0 to 0.4). Symptom duration prior to diagnosis was median 7.5 months (5 to 12). 50% had granulomas present on initial histology. Phenotypes by Paris criteria (Levine et al., 2011) at diagnosis were: B94- colonic, non-stricturing/non-penetrating (L2, B1); B115- colonic, non-stricturing/non-penetrating (L2, B1); B122- ileocolonic, stricturing (L3, B2); B125- ileocolonic, non-stricturing/non-penetrating (L3, B1). This study is publically registered on the United Kingdom Clinical Research Network Portfolio (9633).

#### Biofilm assays

Crystal violet static biofilm assays were performed essentially as described previously (O’Toole, 2011). Briefly, bacteria were grown in RPMI to an OD_600nm_ of 0.6 at 37°C with shaking at 180 rpm. Cultures were further diluted 1:6 before 100 μl was loaded into a 96-well plate, in technical triplicate. The outer wells of the 96-well plate were filled with PBS, only. Plates were sealed with clear plastic seals (Sigma) and placed in a humid chamber. Where indicated, PA was added to a final concentration of 20 mM. Anaerobic culture conditions were achieved using a microaerophilic cabinet. Biofilms were enumerated using the crystal violet method after 5 days of incubation (O’Toole, 2011). All experiments were conducted in biological triplicate.

#### Measurement of 1-^13^C-PA incorporation by gas chromatography coupled to isotope ratio mass spectrometry (GC-C-IRMS)

LF82-PA was grown as before in M9 minimal medium here supplemented with 20 mM 1-^13^C-PA. Cultures were harvested at OD 0.6, pelleted and washed with PBS. Air-dried cells were treated using a saponification and methylation procedure to produce fatty acid methyl esters (FAMEs) of all cell fatty acids. Briefly, to air-dried cells was added 1 mL of methanol:heptane:toluene:2,2-dimethoxypropane:conc H_2_SO_4_ (39:34:20:5:2 by vol) and samples vortexed and then heated at 80°C for 30 mins. Upon cooling, 100 μL of the upper heptane phase containing FAMEs was extracted to a clean vial ready for analysis. Samples were analyzed using gas chromatography coupled to isotope ratio mass spectrometry through a combustion interface (GC-C-IRMS). FAMES separated by GC (Agilent 6890, ZB-FFAP column (30 m x 0.25 mm x 0.25 μm), He carrier (2ml/min), temperature program of 80°C start followed by 7.5°C / min to 150°C, 2°C / min to 225°C and finally 5 min dwell at 225°C) and eluting FAMEs were oxidized to CO_2_ over hot copper oxide (GVI Isochrome, Manchester, UK) in a He flow to the IRMS. An open split design allowed a portion of the eluting CO2 in He to enter the IRMS where ions at mass to charge (*m/z*) 44, 45 and 46 were analyzed continuously and identified peaks were integrated against a reference CO_2_ peak to yield the background and Craig corrected ^13^C/^12^C ratio expressed in the normal units δ13C (per mil) versus the internationally accepted scale for ^13^C/^12^C measurements, VPDB. Samples were bracketed by a certified reference FAME mix (Supelco® 37 Component FAME Mix, Sigma-Aldrich, UK; containing Butyate, Hexanoate, Octanoate, decanoate, Undecanoate, Laurate, tridecanoate, tetradecanoate, Myristoleic, Pentadecanoate, Cis-10-pentadecanoic, Palmitate, palmitoleic, heptadecanoic, cis-10-Heptadecenoic, octadecanoic, trans-9-Elaidic, cis-9-Oleic, Linolelaidic, linoleate, Arachidate, gamma-Linolenic, cis-11-eicosenoate, Linolenate, heneicosanoate, cis-11,14-Eicosadienoic, docosanoate, cis-8,11,14-Eicosatrienoic, Erucate, cis-11,14,17-Eicosatrienoic, tricosanoate, cis-5,8,11,14-Eicosatetraenoic, cis-13-16-Docosadienoic, lignocerate, cis-5,8,11,14,17-Eicosapentaenoate, Nervonate, cis-4,7,10,13,16,19-Docosahexaenoate) to retention time lock for 37 odd and even chain FAMEs.

#### Adherence and invasion assays

Caco-2 IECs were washed once before infection and bacterial suspensions were added at an MOI of 10. Plates were centrifuged after the initial inoculation (700 x *g,* 15 min), before the infection was allowed to proceed for 2 h at 37°C in 5% CO_2_ atmosphere. Non-adhered bacteria were washed away and the infected cells were lysed with 1% Triton X-100 for 5 min. Bacteria were serially diluted in Luria Bertani (LB) broth and spread onto LB agar plates. Total bacteria were enumerated by counting colony forming units (CFUs) after overnight incubation at 37°C. To determine bacterial invasion, cells were infected for 2 h, extracellular bacteria were then washed away and 50 μg/ml gentamycin sulfate was added for 1 h to kill any remaining cell-associated bacteria before Triton X-100 treatment.

#### Gentamicin protection assay

Intracellular replication was analyzed through a gentamicin protection assay over a time course of infection. Bacteria were added at an MOI of 10 to LPS-activated RAW 264.7 cells and the infection allowed to proceed for 1 h. After 1 h, non-internalised bacteria were removed by washing three times in media containing gentamicin (50 μg/ml). Cells were then held in gentamicin containing media for 2 h. After 2 h (time point = 0h), cells were washed three times in PBS, before being lysed with 1% Triton X-100. Bacteria were enumerated via serial dilution. A further time point at 4 h post the 0 h time point was analyzed.

#### Acid survival assays

Cultures of bacteria were grown overnight at 37°C in LB. The pH of these cultures was lowered to pH 3 using 1 M HCl. Samples were taken every 20 min for 1 h and serially diluted in LB. Dilutions were plated in triplicate onto LB agar and incubated overnight at 37°C. Colonies were counted to determine the number of surviving cells.

#### Total RNA extraction and mRNA enrichment

Cultures were grown overnight in LB, washed three times in NCE media with no carbon source added, and inoculated 1:100 into 10 mL NCE media containing each respective carbon source. Cultures were grown until mid-log phase (OD600 of 0.6) and mixed with two volumes of RNAprotect reagent (QIAGEN, Valencia, CA, USA), before incubating for 5 min at room temperature. Total RNA was extracted, genomic DNA removed and samples enriched for mRNA as described previously by Connolly et al. (2016). Samples for RNA-sequencing (RNA-seq) analysis were QC tested for integrity and rRNA depletion using an Agilent Bioanalyzer 2100 (University of Glasgow, Polyomics Facility).

#### Genomic analysis and SNP identification

A bacterial lawn generated from single overnight colonies of LF82 and three independent cultures of LF82-PA were resuspended in a microbank bead tube, inverted four times and incubated at room temperature for 2 min. The cryopreservative was removed and the samples sent to MicrobesNG (Birmingham University, UK) for sequencing. Genomic DNA was extracted using a Illumina Nextera XT DNA sample kit as per manufacturer’s protocol (Illumina, San Diego, USA). Samples were sequenced on the Illumina MiSeq using a 2x250 paired-end protocol, De novo assembled using SPAdes version 3.5, aligned to the reference genome using BWA-MEM 0.7.5. Variants were called using samtools 1.2 and VarScan 2.3.9 and annotated using snpEFF 4.2. Subsequent genomic analysis was performed using a combination of MAUVE, CLC genomics (Version 7.0.1), ExPASY and EMBOSS Needle. The sequence reads in this paper have been deposited in the European Nucleotide Archive under accession number PRJEB36206.

#### RNA-seq transcriptome generation and data analysis

cDNA synthesis and sequencing was performed at the University of Glasgow Polyomics Facility, essentially as described by Connolly et al. (2016). Briefly, sequencing was preformed using an Illumina NextSeq 500 platform obtaining 75 bp single end reads. Samples were prepared and sequenced in triplicate. Raw reads were QC checked using FastQC (Babraham Bioinformatics, Cambridge, UK) and trimmed accordingly using CLC Genomics Workbench (CLC Bio, Aarhus, Denmark). Trimmed reads were mapped to the LF82 reference genome (NCBI accession number: CU651637) allowing for 3 mismatches per read. Analysis of differential expression was performed using the Empirical analysis of DGE tool, which implements the EdgeR Bioconductor tool (Robinson et al., 2010). Differentially expressed genes were identified by absolute fold change (cutoffs log_2_) and a P value of ≤ 0.05. Volcano plots were generated in CLC Genomics Workbench. The sequence reads in this paper have been deposited in the European Nucleotide Archive (PRJEB36206).

#### Quantitative real-Time PCR (qRT-PCR)

cDNA was generated from total RNA using an Affinity Script cDNA Synthesis Kit (Agilent) following the manufacturer’s instructions. Levels of transcription were analyzed by qRT-PCR using PerfeCTa® SYBR® Green FastMix® (Quanta Biosciences). Individual reactions were performed in triplicate within each of three biological replicates. The 16S rRNA and *rpoS* genes were used to normalize the results. RT-PCR reactions were carried out using the ECO Real-Time PCR System (Illumina, San Diego, CA, USA) according to the manufacturer’s specifications and the data were analyzed according to the 2^-ΔΔCT^ method (Livak and Schmittgen, 2001). All primers used are listed in Table S1.

##### Construction of p16Slux

LF82 and LF82-PA *lux* integrated strains containing the erythromycin cassette were generated using the protocol of Riedel et al. (2007). The bioluminescent properties of these strains allowed visualization of the establishment of infection but despite bacteria being recovered it was noted that bioluminescent signal was lost. However, upon plating the murine microbiome onto LB agar containing ampicillin (100 μg/ml), it was observed that several members of the microbiota also harbored ampicillin resistance. LB supplemented with erythromycin (500 μg/ml) did not support the growth of any microbiota species; therefore utilizing the erythromycin cassette inserted as part of the *lux* integration allowed for the selection of LF82 and LF82-PA, and was used in subsequent animal experiments. Strains containing the *lux* cassette were only used during *in vivo* infections and subsequent *in vitro* experiments when these strains were re-isolated from the murine intestine and tested for virulence.

#### SCFA analysis by gas chromatography

Faecal contents of murine caeca were isolated from PBS treated mice three dpi. The concentrations of acetate, propionate and butyrate per gram of dry weight were measured by gas chromatography as previously described (Laurentin and Edwards, 2004) and expressed as a ratio for comparison to the known human acetate:propionate:butyrate SCFA ratio (Cummings et al., 1987). The effect of PA supplementation in drinking water on PA levels was measured by extraction of caecal contents from PA treated and untreated mice and the levels of SCFAs again calculated per gram of dry weight.

### Quantification and Statistical Analysis

Values are represented as means and standard deviation. All statistical tests were performed with GraphPad Prism software, version 7.0c. All replicates in this study were biological; that is, repeat experiments were performed with freshly grown bacterial cultures, immortalized cells and additional mice, as appropriate. Technical replicates of individual biological replicates were also conducted, and averaged. Significance was determined as indicated in the figure legends. RT-PCR data was log-transformed before statistical analysis. Values were considered statistically significant when *p-value*s were *^∗^p < 0.05; ^∗∗^p < 0.01; ^∗∗∗^p < 0.001; ^∗∗∗∗^p < 0.0001*.

### Data and Code Availability

The sequence reads in this paper have been deposited in the European Nucleotide Archive (ENA:PRJEB36206).

## Supplemental Information

Document S1. Figures S1–S5 and Tables S1 and S2

Data S1. Sequence Analysis of Reference Strain LF82, Related to Figure 3

Document S2. Article plus Supplemental Information
