# Supplementary material for: Propionic Acid Promotes the Virulent Phenotype of Crohn’s Disease-Associated Adherent-Invasive Escherichia coli
Source: Cell Rep. 2020 Feb 18;30(7):2297–2305.e5. doi: 10.1016/j.celrep.2020.01.078 (PMC7034058; doi:10.1016/j.celrep.2020.01.078)
Supplement: Document S1. Figures S1–S5 and Tables S1 and S2 [file mmc1.pdf]

Cell Reports, Volume *30*

Supplemental Information

Propionic Acid Promotes the Virulent Phenotype of Crohn's Disease-Associated Adherent-Invasive *Escherichia coli*

Michael J. Ormsby, Síle A. Johnson, Nuria Carpena, Lynsey M. Meikle, Robert J. Goldstone, Anne McIntosh, Hannah M. Wessel, Heather E. Hulme, Ceilidh C. McConnachie, James P.R. Connolly, Andrew J. Roe, Conor Hasson, Joseph Boyd, Eamonn Fitzgerald, Konstantinos Gerasimidis, Douglas Morrison, Georgina L. Hold, Richard Hansen, Daniel Walker, David G.E. Smith, and Daniel M. Wall

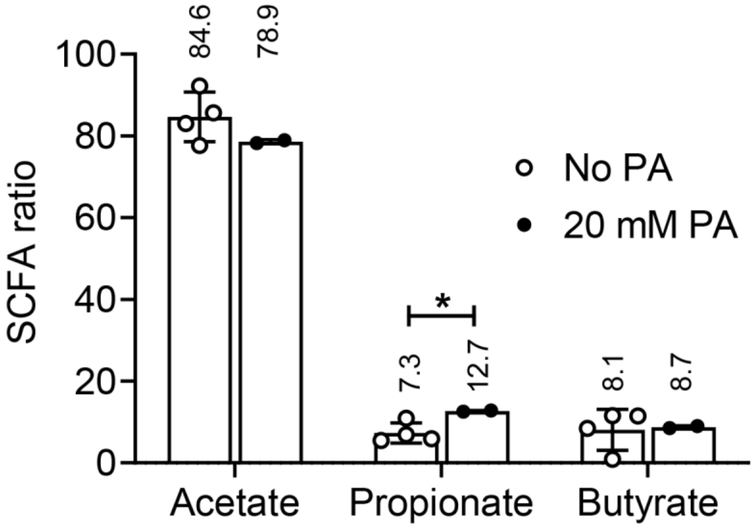

Figure S1 Dietary supplementation with PA alters the SCFA ratio of the murine intestine, related to Figure 1. Levels of murine caecal SCFAs were determined by dry weight before and after supplementation of the drinking water with 20mM PA. Levels were converted to ratios. Actual numbers are displayed above each bar. Data were analysed by two-tailed unpaired t-test with Welch's correction where $p<0.05$ *.

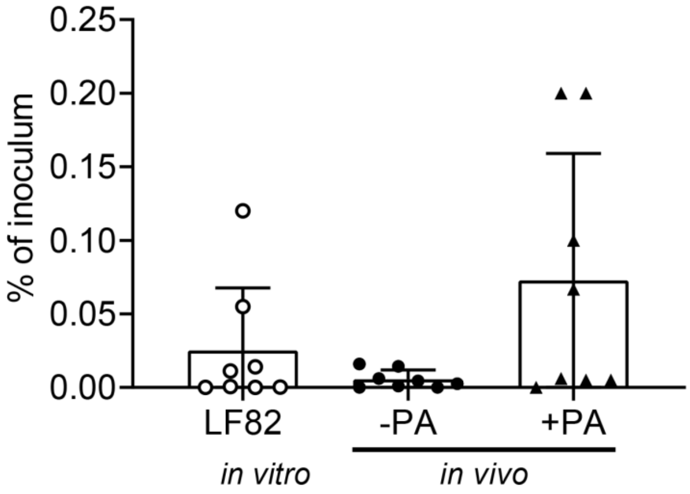

Figure S2 *In vivo* exposure of AIEC to PA increases *ex vitro* invasion of intestinal epithelial cells, related to Figure 1. Invasion of caco-2 intestinal epithelial cells was assessed. *In vitro/vivo* refers to where the strains were generated. AIEC type strain LF82 modified to contain the luciferase and erythromycin cassette (LF82*lux*) was recovered from mice that had been given water (-PA) or water supplemented with 20 mM propionic acid (+PA). Samples were analyzed using a one-way ANOVA with Holm-Sidaks multiple comparisons post-test.

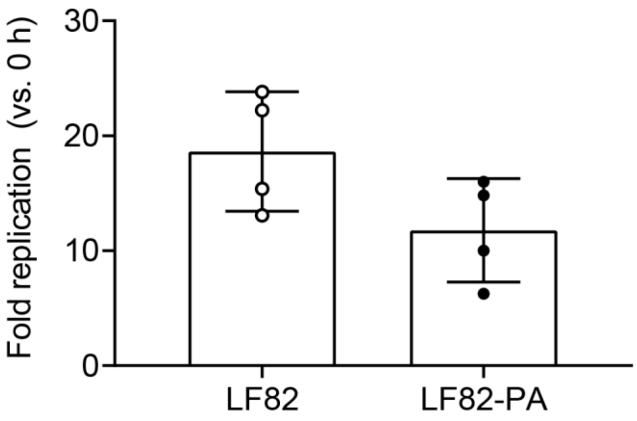

Figure S3 LF82 adaptation to PA does not affect intracellular replication, related to Fig. 2. The ability of LF82 and LF82-PA strains to replicate intracellularly was determined. Results displayed as fold differences 4h post infection. Results displayed are the average of at least three biological replicates ± SD. Samples were analyzed by two-tailed unpaired t-test with Welch’s correction.

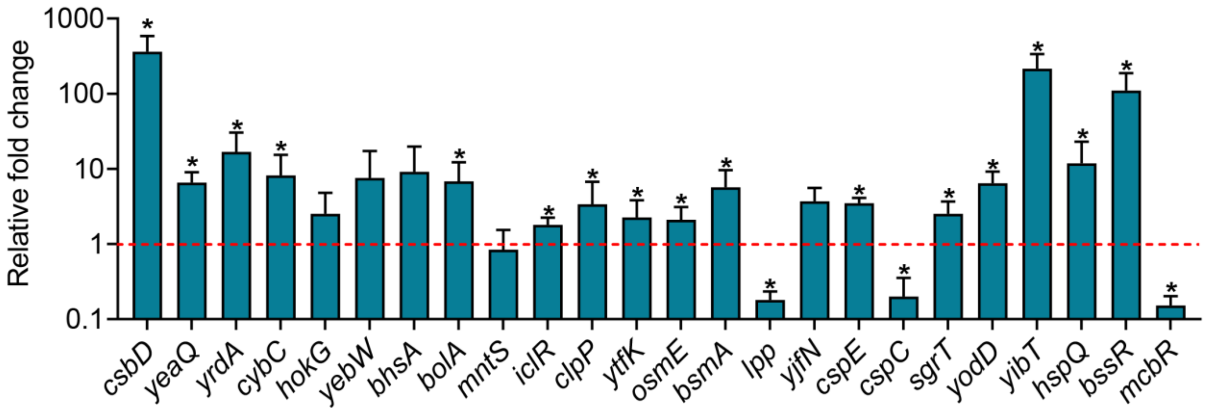

Figure S4 qRT-PCR validation of transcriptional changes of LF82-PA, related to Fig. 4. qRT-PCR was conducted on LF82-PA grown in minimal media supplemented with 20 mM PA. Relative fold change was measured against LF82 grown under the same conditions, using *16S* as a control. Three independent biological replicates were performed. Data are expressed as relative fold change ± SD and were analyzed using a one-way ANOVA with Tukey; $p<0.05$ *.

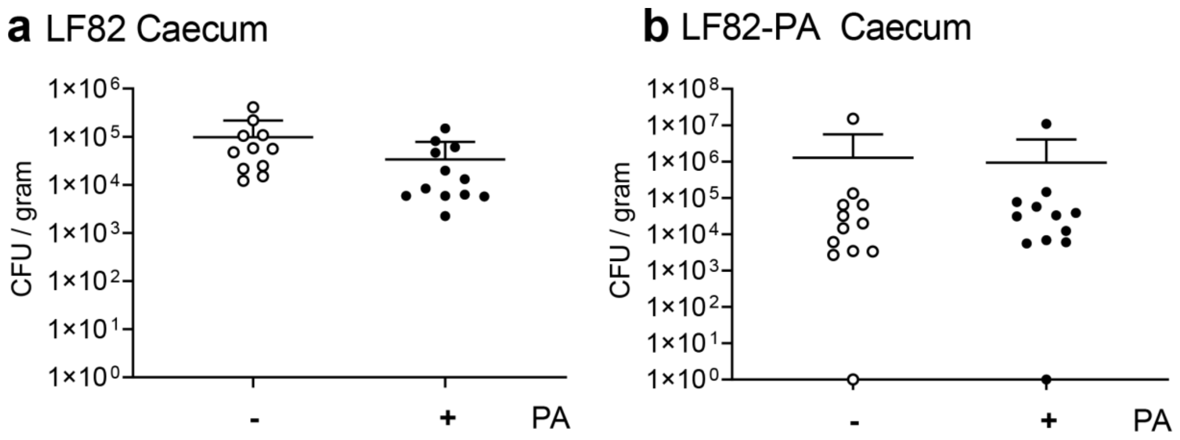

Figure S5 Exogenous PA supplementation does not alter colonization and long-term persistence of AIEC in the murine caecum, related to Fig. 5. Drinking water was supplemented where indicated with 20mM PA and provided to male C57BL/6 mice for three days prior to infection. Persistence was determined 21 days post infection by colony counts. Data are expressed as CFU/gram of tissue ± SD and were analysed using an unpaired students t-test; ns=not significant.

Table S1 ***List of oligonucleotides used in this study,*** **related to Figure 4 and Figure S4.**

Primer	Sequence 5' - 3'
16s rRNA_For	TGAGAATGTGCCTTCGGGAG
16s rRNA_Rev	TTGCGGGACTTAACCCAACA
BhsA_For	GGGACAAATCTGGGATCGCT
BhsA_Rev	CCATGGAGGGTATTCGGACC
BolA_For	GCGTTCCAACCCGTATTCCT
BolA_Rev	ATCGCTGACCAGCACAACTT
BsmA_For	TCTCCGGATGACGCATTAGC
BsmA_Rev	ACCACTGTCCTGTCACGATG
BssR_For	GCGAATCGATCTGCTGAACC
BssR_Rev	GATTGCTTTCGCTGACGGAC
ClpP_For	AACCTGATTGTGGCGCAGAT
ClpP_Rev	CCCCGCCTGGAGAGTTAATG
CsbD_For	GGTAAACGTGATCAACTGGTCG
CsbD_Rev	ATCATGGCGTTTTTCCCAACT
CspC_For	CCGCTATCCAGGGTAATGGC
CspC_Rev	TAACAGCTGCCGGACCTTTC
CspE_For	AAGACGGCAGCAAAGACGTG
CspE_Rev	TGATTTCGAACTCTACGCGCT
CybC_For	AACTAAAATGCGCGCTGCGG
CybC_Rev	AGTCTTTCATTTCCGGGCTGT
EutS_For	GCGCAGTAGAAGAAGCGTT
EutS_Rev	TTGGTCATTTCACAAAGCGT
HokG_For	TGCTGACAAAATATGCCCTTGTG
HokG_Rev	CAACTCACACAACGAGTCGC
HspQ_For	CGACCCGGTTTATTCGCTCT
HspQ_Rev	TAGGCCGTTATCGTCCTCCA
IclR_For	AGCATGTTGTCGGCCTGAAT
IclR_Rev	TTGATGAAGGCCCGGAGATG
Lpp_For	CAGCTGAGCAACGACGTGAA
Lpp_Rev	GTAGCCATGTTGTCCAGACG
McbR_For	GGATCCGCTACGAACTGGAA
McbR_Rev	ATCGGCTGTATTGTTGGCAC
MntS_For	GGTGTATGCGCGTGTTTAGTC
MntS_Rev	AAGGTTTATCCTGCTGCGGT
OsmE_For	ATCCTGGGTCAACGTGATGG
OsmE_Rev	GCACAGGTCTGATAACCGGA
PduA_For	AGGCTTAACTGCCGCCATAG

Primer	Sequence 5' - 3'
PduA_Rev	CACCAGCCCGGAACCTATTT
PrpB_For	TATTCGCCGTATCACCGACG
PrpB_Rev	CGCCACGTTAAAGGCTGAAG
Pta_For	GCTGAAGTCGTTCTGGTGGA
Pta_Rev	TTCTGCGTTCAGCGTTTTGG
SgrT_For	CAGCGAAGTTGTGCTGGTTG
SgrT_Rev	TCAGAATGACTCCCCTCCCA
TdcD_For	CACTGGCACCCCTGCATAAT
TdcD_Rev	TCAAATACCGCCACCTGAGT
YeaQ_For	GATAGTCGGTGCCGTAGTCG
YeaQ_Rev	CAACCACGAAGCTGCCAAAA
YebW_For	ACACGGAACAGCAGTGTCTT
YebW_Rev	CAGGTCGCCAGAAACCATCT
YibT_For	GGTGAAAACGTTCCGCTTCT
YibT_Rev	GCGTTACGGGGAGGTAGTTT
YjfN_For	GGACGTGGAACGTATCGTGG
YjfN_Rev	TATCAGGCTGCTGGTCTTCG
YodD_For	AAAACGTGAGGTCAGCGTCG
YodD_Rev	TCGACGCTAACGTGTTCAGA
YrdA_For	TACAACCCAGATGGCAACCC
YrdA_Rev	CTCGATTGCCAATGGTGCAG
YtfK_For	TCCAACGGTACAACCCACTT
YtfK_Rev	ATTCAAAAGCGCCAACGTCC

Table S2 ***RPKM values of differentially expressed genes following RNA-seq*****, related to Figure 4.**

Gene	RPKM(log_2)			Function	Reference
	LF82-PA.1	LF82-PA.2	LF82-PA.3		
csbD	1.263	1.189	1	Stress response - overexpressed in virulent *E. coli.*	Amigo et al., 2016
yeaQ	1.0144	0.9486	0.1763	Function unknown.	-
yrdA	0.856	1.5558	-2	Carbonic anhydrase-like. Catalyze the rapid interconversion of CO_2 and H_2O to HCO_3	Merlin et al., 2003
cybC	0.9635	0.5753	0.5361	Electron-transport protein of unknown function.	-
bssS	0.5656	0.585	2.1342	Induced in biofilms. Motility regulation.	Domka et al., 2006
hokG	2.5261	-0.6666	2.1953	Toxin/Antitoxin system	
yebW	1.9745	1.3219	1.546	Function unknown.	-
bhsA (ycfR)	1.2869	2.4803	2.1177	Induced in biofilms. Stress response.	Zhang et al., 2007
bolA	4.3067	4.4209	4.6753	Switch from motility to biofilms.	Dressaire et al., 2015
mntS	4.0522	4.0959	3.1194	Optimize the Intracellular Concentration of Manganese	Martin et al., 2015
iclR	5.8071	6.1208	5.6424	Isocitrate lyase regulator. Repressor of *aceBAK* operon for glyoxylate shunt.	Molina-Henares et al., 2006
clpP	2.9373	2.6206	2.2357	Protease. Stress tolerance, biofilm formation and intracellular invasion.	Xie et al., 2013
ytfK	3.6041	4.591	4.0807	Regulated by *phoB*. Function unknown.	Baek and Lee, 2006
osmE	3.0942	2.7027	3.0461	Response to osmotic stress.	Gutierrez et al., 1995
bsmA (yjfO)	5.5163	5.3466	5.0492	Biofilm formation and stress response.	Weber et al., 2010
lpp	6.7733	5.9896	6.5633	Provides structural integrity to the outer membrane.	
yjfN	3.9964	4.0295	4.1506	Unknown. Downstream of *bsmA* (*yjfO*).	Weber et al., 2010
cspE	10.9825	10.4866	10.3823	Roles in membrane stress, motility, virulence and biofilm formation.	Michaux et al., 2017
cspC	3.5097	3.3937	2.7377	Roles in membrane stress, motility, virulence and biofilm formation.	Michaux et al., 2017

Gene	RPKM(log_2)			Function	Reference
	LF82-PA.1	LF82-PA.2	LF82-PA.3		
sgrT	6.8381	7.4463	6.4256	Increased in biofilms. Inhibits glucose use and encourages the utilisation of alternative carbon sources.	Raina and Storz, 2017
yodD	5.8432	5.8802	5.3847	Hydrogen peroxide, cadmium and acid stress	Lee et al., 2010
yibT	4.1683	4.9141	4.5033	Regulating membrane fatty acid composition.	Si et al., 2016
hspQ	4.9667	5.0259	4.4899	Stress response.	Puri et al., 2017
bssR	8.9034	9.1047	8.4232	Induced in biofilms. Motility regulation.	Domka et al., 2006
mcbR	-16.6096	-16.6096	-16.6096	Regulator of biofilm formation.	Zhang et al., 2008
